# Deposition of Polymer Particles with Fibrinogen Corona at Abiotic Surfaces under Flow Conditions

**DOI:** 10.3390/molecules26206299

**Published:** 2021-10-18

**Authors:** Paulina Żeliszewska, Monika Wasilewska, Michał Cieśla, Zbigniew Adamczyk

**Affiliations:** 1 Jerzy Haber Institute of Catalysis and Surface Chemistry Polish Academy of Science, Niezapominajek 8, 30-239 Krakow, Poland; monika.wasilewska@ikifp.edu.pl; 2 Faculty of Physics, Astronomy, and Applied Computer Science, Jagiellonian University, Stanisława Łojasiewicza 11, 30-348 Krakow, Poland; michal.ciesla@uj.edu.pl

**Keywords:** adsorption of fibrinogen, deposition of particles with corona, fibrinogen corona at particles, microfluidic impinging-jet cell, zeta potential of protein coronas

## Abstract

The deposition kinetics of polymer particles with fibrinogen molecule coronas at bare and poly-L-lysine (PLL) modified mica was studied using the microfluid impinging-jet cell. Basic physicochemical characteristics of fibrinogen and the particles were acquired using dynamic light scattering and the electrophoretic mobility methods, whereas the zeta potential of the substrates was determined using streaming potential measurements. Subsequently, an efficient method for the preparation of the particles with coronas, characterized by a controlled fibrinogen coverage, was developed. This enabled us to carry out measurements, which confirmed that the deposition kinetics of the particles at mica vanished at pH above 5. In contrast, the particle deposition of PLL modified mica was at maximum for pH above 5. It was shown that the deposition kinetics could be adequately analyzed in terms of the mean-field approach, analogously to the ordinary colloid particle behavior. This contrasts the fibrinogen molecule behavior, which efficiently adsorbs at negatively charged substrates for the entire range pHs up to 9.7. These results have practical significance for conducting label-free immunoassays governed by the specific antigen/antibody interactions.

## 1. Introduction

Protein immobilization of carrier particles is essential for their separation and purification by filtration, for biosensing, enzymatic catalysis, bioreactors, immunological assays, etc. [1,2,3,4]. In the case of nanoparticle carriers, a controlled protein attachment leads to the “corona” formation extensively studied for single-molecule systems and for mixtures comprising the blood serum [5,6,7,8]. However, because of relatively low stability, nanoparticles decorated by coronas are difficult to study by conventional techniques, which require suspension centrifugation or filtration.

In contrast, a physical (non-specific) immobilization of protein molecules at particles of larger size, for example, polymer microspheres (latexes), is advantageous because such conjugates show larger stability than the protein solution themselves. Additionally, investigations of such systems can furnish information about the protein behavior in bulk solutions, for example, about their isoelectric point, even if minor amounts of proteins are available [9,10,11,12]. Acquiring protein physicochemical characteristics in this way is of special interest for expensive proteins such as immunoglobulins, which are currently used in a plethora of sensitive agglutination immunoassays [12,13], and for spike proteins that control virus particle attachment to abiotic surfaces or to cell membranes.

Given its significance, protein molecule adsorption at polymer particles was often studied in the literature, mainly using electrophoretic mobility measurements and concentration depletion methods [9,12,13,14,15,16,17,18,19,20,21,22]. A quantitative interpretation of these experimental results was carried out in terms of an electrokinetic model representing the extension of the Smoluchowski approach for heterogeneous (particle covered) surfaces. This enabled to control the protein coverage at polymer carrier particles via simple electrophoretic mobility measurements [9,10,11]. Moreover, the orientation of adsorbed molecules can be regulated by pH, ionic strength, and by the surface charge of the particles [22].

However, despite the significance of the protein/microparticle conjugates for biosensing, few experimental investigations focused on the kinetics of their deposition at solid substrates were reported. The adsorption kinetics of fibrinogen/polystyrene microparticle conjugates on mica and silica was studied under diffusion-controlled transport, which is a tedious and rather inflexible procedure [23].

One may expect that flexible and practically important experiments can be performed applying microfluidic flow cells, which create the possibility of direct in situ measurements of particle deposition kinetics on various substrates [24,25]. However, up to our knowledge, experimental data pertinent to particles with protein coronas have not been reported in the literature. Therefore, in view of the lack of adequate information, the goal of this work is to elucidate mechanisms of such particle deposition on mica, representing a model negatively charged substrate and poly-L-lysine (a cationic macroion) modified mica. The latter represents a model biocompatible substrate characterized by a positive charge. The role of pH is thoroughly investigated in order to select appropriate conditions for the irreversible physical attachment of the particles to these substrates. Experiments are performed in the oblique impinging-jet cell (OBJ) [25], allowing real-time and in situ observations of particle trajectories and deposition/desorption events under well-controlled and regulated flow conditions.

In our work, attention is focused on fibrinogen, which can be considered as a model antigen. It is an abundant blood plasma glycoprotein that plays an essential role in the clotting cascade, platelet adhesion, thrombosis, angiogenesis, inflammatory response, tumor growth, and fouling of implants, etc. [26,27,28]. The fibrinogen molecule is composed of two symmetric parts, each consisting of three different polypeptide chains named Aα, Bβ, and γ, that are joined together by disulfide bonds [29,30,31,32]. A major part of the Aα chains extends from the core of the molecule, which forms two appendages, each having a molar mass equal to 42.3 kg mol^−1,^ whereas the entire molecule molar mass is equal to 338 kg mol^−1^ [32]. The net charge of the molecule derived from electrophoretic mobility measurements [33] is positive for pH below 5.8 that is considered as its isoelectric point. It is worth underlining that the charge distribution over the fibrinogen molecule is heterogenous, with the core part being negative at pH larger than four and the Aα chains being positive at pH up to ten [33]. This property is advantageous because it creates the possibility to control adsorbed molecule orientation adsorbed at various substrates by their surface charge, pH, and ionic strength. [34] The Horvath group [35,36] performed interesting measurements for the bacterial protein flagellin, whose molecule, similarly to fibrinogen, exhibits an elongated shape. The results obtained in these experiments showed that the flagellin molecules mostly adsorb in the end-on orientation.

It is expected that the obtained results can be significant to basic science, furnishing new quantitative information about deposition mechanisms of polymer particles with a protein corona at substrates characterized by different surface charge. This knowledge can be used for predicting the kinetics of bioparticle, for example, virus deposition on abiotic surfaces. In addition, the acquired results can be exploited for devising robust biosensing assays and for a quantitative calibration of cells used in flow cytometry techniques [36].

## 2. Results and Discussion

### 2.1. Physicochemical Characteristics of Fibrinogen and Substrates

**Figure 1:**
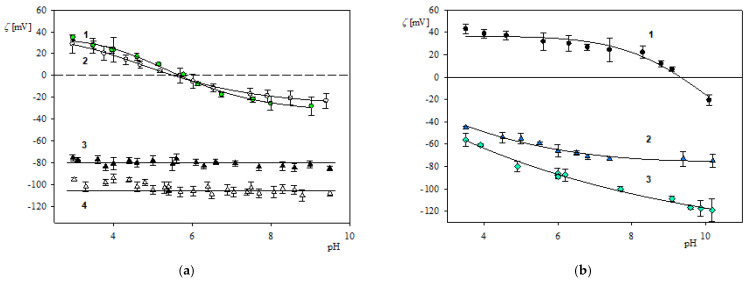
Part (**a**). The dependence of the zeta potential of fibrinogen molecules and LS particles on pH determined by the LDV method. 1—fibrinogen, 0.001 mol L^−1^ NaCl, 2—fibrinogen, 0.01 mol L^−1^ NaCl, 3—LS particles, 0.001 mol L^−1^ NaCl, 4—LS particles, 0.01 mol L^−1^ NaCl; Part (**b**). The dependence of the zeta potential of mica on pH determined by the streaming potential method. 1—mica/PLL layer, 0.01 mol L^−1^ NaCl, 2—bare mica, 0.01 mol L^−1^ NaCl, 3—bare mica, 0.001 mol L^−1^ NaCl. The solid lines represent fits of experimental data.

The electrokinetic characteristics of fibrinogen molecules, the polymer particles, and the solid substrates comprising bare and PLL modified mica were acquired accordingly to the above-described procedures. Primarily, the electrophoretic mobility *μ_e_* as a function of pH was directly measured for different ionic strengths using the LDV method (Supporting Information). These results were converted to the zeta potential vs. pH dependencies and are presented in Figure 1a. As can be seen, the zeta potential of fibrinogen molecules at pH 3.5 is equal to 36 and 28 mV, for 0.001 and 0.01 mol L^−1^ NaCl concentration, respectively. It systematically decreases vanishing at pH 5.8 (isoelectric point) and attains negative values at larger pHs.

The zeta potential of the polymer particles was determined in an analogous way via the electrophoretic mobility measurements. For polystyrene particles (LS) it was equal to −78 ± 5 and −105 ± 6 mV at pH 3.5 and NaCl concentration of 0.01–0.001 mol L^−1^, respectively (see Figure 1a). For the amidine (LA) particles at pH 3.5, the zeta potential was equal to 74 ± 4 and 85 ± 6 mV (for 0.01 and 0.001 mol L^−1^ NaCl).

To complete the particle characteristics, their size distribution was determined from the Stokes–Einstein formula exploiting the diffusion coefficient values acquired by DLS measurements. Thus, the hydrodynamic diameter of the LS particles at pH range 3.5–9 was equal to 850 ± 20 and 820 ± 15 nm for 0.001 and 0.01 mol L^−1^ NaCl, respectively, whereas the hydrodynamic diameter of the LA particles at the pH the range 3 to 10 and NaCl concentration of 0.001 to 0.01 mol L^−1^ was equal to 810 ± 20 nm.

On the other hand, the zeta potential of bare and PLL modified mica was determined by the streaming potential measurements as described in the experimental and methods section. It is graphically presented in Figure 1b as a function of pH. In the case of the bare mica, the zeta potential decreases from−45 mV at pH 3.5 to −70 mV at pH 10 (for the NaCl concentration of 0.01 mol L^−1^). The zeta potential for the PLL modified mica was equal to 40 and 25 mV at pH 3.5 and 7.4, respectively (0.01 mol L^−1^ NaCl). At pH larger than 9.5, the zeta potential became negative.

### 2.2. Formation of Fibrinogen Corona at Polymer Particles

Formation of fibrinogen coronas was carried out according to the previously described procedure [9] by mixing equal volumes of microparticle suspension of the concentration *c_p_* (typically equal to 100 mg L^−1^) with fibrinogen solution of the concentration *c_f_* varied between 0.1–5.0 mg L^−1^. After the adsorption time of 900 s, the electrophoretic mobility of the particles with fibrinogen corona, hereafter referred to as LSfi particles, was measured under static conditions using the LDV method. Finally, the zeta potential of particles was calculated using the Smoluchowski formula. It should be mentioned that the characteristic time of fibrinogen corona formation for the above particle concentration is equal to a few seconds [9].

Primary results obtained in the above experiments are expressed as the dependence of the zeta potential of the LSfi particles on the nominal fibrinogen corona coverage *Γ* calculated from the formula

*Γ* = *v*_s_·*c_f_*/*S_s_* (1)

where *v*_s_ is the volume of the mixture, *c_f_* is the fibrinogen concentration in the suspension after mixing with the particles, and *S_s_* is the surface area of the bare particle suspension.

**Figure 2:**
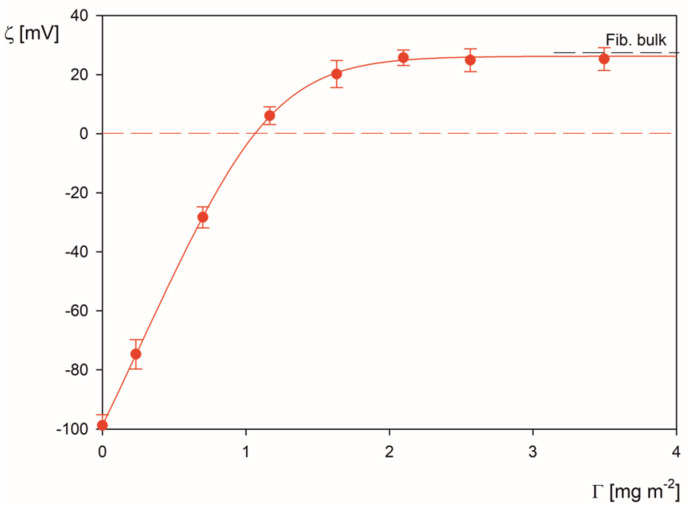
Zeta potential of the LS particles vs. the nominal coverage of the fibrinogen corona *Γ*; pH 3.5, 0.01 mol L^−1^ NaCl. The points denote experimental results obtained for the particle concentration of 100 mg L^−1^ using the LDV method, and the solid line shows the theoretical results calculated from the electrokinetic model.

The results obtained for 0.01 mol L^−1^ NaCl and pH 3.5 and shown in Figure 2 indicate that the zeta potential of the polymer particles abruptly increases with the corona coverage and becomes positive for *Γ* > 1.2 mg m^−2^. For still larger corona coverage, the changes in the zeta potential become minor, and finally, the limiting value of the zeta potential equal to 26 mV is attained, which is close to the bulk zeta potentials of fibrinogen molecules equal to 28 mV. One can estimate that the maximum corona coverage, where the zeta potential of microparticles ceases to change, is approximately equal to 2.5 mg m^−2^ for the NaCl concentration of 0.01 mol L^−1^. However, given the limited precision of the maximum coverage determination by the direct LDV measurements, a more precise concentration depletion method was applied to derive a more accurate value of the coverage [9]. Accordingly, the residual concentration of fibrinogen in the suspension after the corona formation step was quantitatively determined by the adsorption at mica sheets under a diffusion-controlled regime. Single fibrinogen molecules were imaged by AFM and their surface concentration was determined by a direct counting procedure [9]. In this way, the threshold concentration of fibrinogen could be determined where it starts to appear on mica sheets. Knowing this concentration, the maximum coverage of the corona can be calculated from Equation (1). It is determined in this way that the maximum corona coverage of irreversibly adsorbed fibrinogen molecules was equal to 2.6 ± 0.2 mg m^−2^ and 2.1 ± 0.2 mg m^−2^ (for 0.01 and 0.001 mol L^−1^ NaCl, respectively, and pH 3.5).

The experimental data shown in Figure 2 were interpreted using the electrokinetic model where the three-dimensional fluid velocity and electric potential distributions around protein molecules forming the corona are considered. This approach makes it possible to calculate the zeta potential of the particles with the corona if the fibrinogen coverage, its bulk zeta potential, and the zeta potential of the bare LS microparticles are known (see Supporting Information). One can observe in Figure 2 that the theoretical results derived from this model (shown as the solid line) adequately reflect the experimental data. This observation, which confirms that the zeta potential of particles with protein coronas strictly correlates with the bulk zeta potential of the protein molecules, has significant implications for the prediction of coronavirus zeta potentials. This is especially important for the SARS-Cov-2 virus, where the spike protein zeta potential can only be experimentally determined for its recombinant version, in contrast to the intact virion where the zeta potential measurements are impractical.

**Figure 3:**
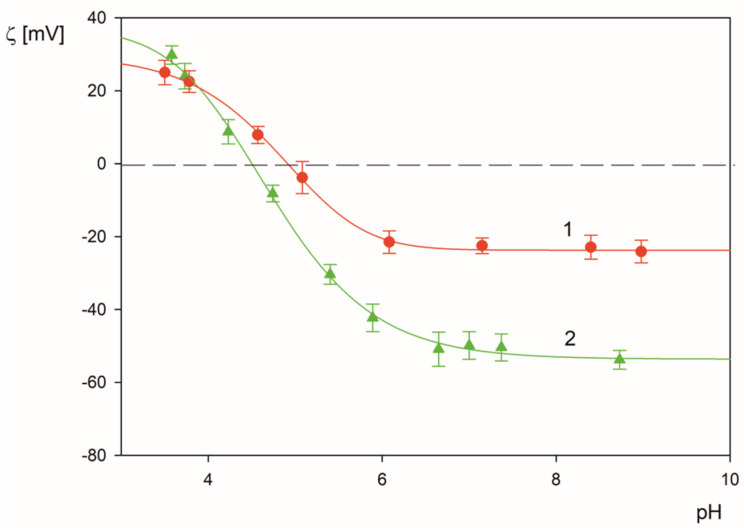
The dependence of the zeta potential of the LSFi particles on pH (fibrinogen corona of the coverage equal to 2.2 mg m^−2^ was formed at pH 3.5 and 0.01 mol L^−1^ NaCl). The points represent experimental data acquired using the LDV method for (●) 0.01 mol L^−1^ NaCl, (▲) 0.001 mol L^−1^ NaCl. The solid lines 1, 2 are fits of experimental data.

It was also determined that the LSFi particle suspensions were stable for pH range 3 to 9 over the time exceeding 24 h (see Supporting Information). This facilitated determination of their zeta potential, as a function of pH and ionic strength, for various corona coverage. Results of such measurements, performed for the corona coverage of 2.2 mg m^−2^ are presented in Figure 3. As can be seen, the zeta potential of the LSFi particles vanishes at a pH approximately equal to 5, which is lower than the isoelectric point of the fibrinogen molecules in the bulk, equal to 5.8 [22]. It should be mentioned that such a shift of isoelectric point to more acidic pHs of protein monolayers adsorbed at polymer carrier particles was reported in the literature [37,38]. Furthermore, a similar behavior was observed for various coronaviruses [39] where the zeta potential of the intact virion (derived from electrophoretic mobility measurements) vanished at pH 4. In contrast, the theoretically calculated charge from the crystallographic structure of the virion was positive for pH up to 9, hence, the virion showed no isoelectric point.

**Figure 4:**
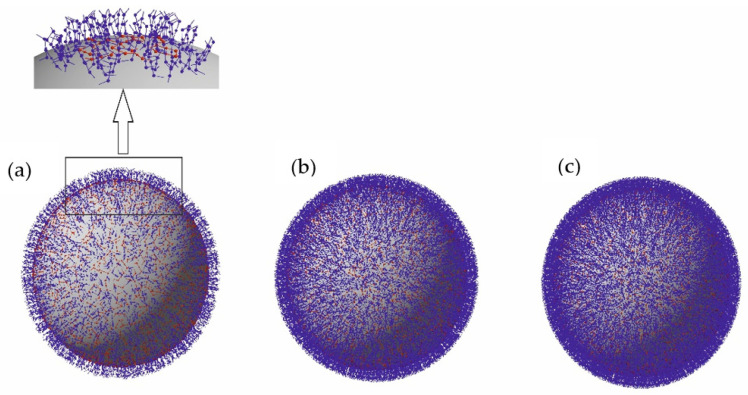
The fibrinogen coronas at LS particles derived from the RSA modeling for 0.01 mol L^−1^ NaCl, pH 3.5 (simultaneous side-on and end-on adsorption). (**a**) -*N* = 1550 µm^−2^ (*Γ* = 0.87 mg m^−2^ ), (**b**) -*N* = 3900 µm^−2^ (*Γ* = 2.2 mg m^−2^ ), (**c**) -*N* = 5100 µm^−2^ (*Γ* = 2.8 mg m^−2^ ), the molecules adsorbed side-on are marked in red and the end-on adsorbed molecules are marked in blue.

In order to quantitatively interpret the fibrinogen corona formation mechanisms, extensive theoretical modeling was performed according to the algorithm described in the Theoretical Modeling Section. It is determined that the maximum coverages are equal to 0.56, and 3.2 and 2.8 mg m^−2^ for the side-on, end-on, and the mixed regimes, respectively (at pH 3.5 and 0.01 mol L^−1^ NaCl). Moreover, it is calculated that in the latter case, the fractions of side-on and end-on adsorbed molecules were equal to 0.2 and 2.6 mg m^−2^, respectively. Thus, the maximum coverage predicted for the mixed adsorption regime agrees with the experimental result within error bounds. This suggests that the orientation of fibrinogen in the corona at polymer particles is mostly end-on analogously to the spike protein orientation in the capsid of the SAR-Cov-2 virus [40]. A preferred end-on orientation of fibrinogen molecules is well visible in Figure 4, where snap-shots of fibrinogen coronas on polymer particles for various surface concentrations are shown.

The prevailing end-on orientation is further confirmed by the DLS measurements of the diffusion coefficient of the LSFi particles that allowed the determination of their hydrodynamic diameter. At pH 3.5 and 0.01 mol L^−1^ NaCl the hydrodynamic diameter of the LSFi particles was equal to 920 nm for the fibrinogen corona coverage of 2.2 mg m^−2^ compared to the value of 820 nm for bare particles. This suggests that the fibrinogen monolayer thickness is about 50 nm, which almost matches the length of the core part of the fibrinogen molecule used in the theoretical modeling equal to 48.7 nm (see Supporting Information). Hence, the experimental data confirms that the fibrinogen molecules in the corona are preferentially adsorbed in the end-on orientation.

### 2.3. Particle Deposition at Solid Substrates

**Figure 5:**
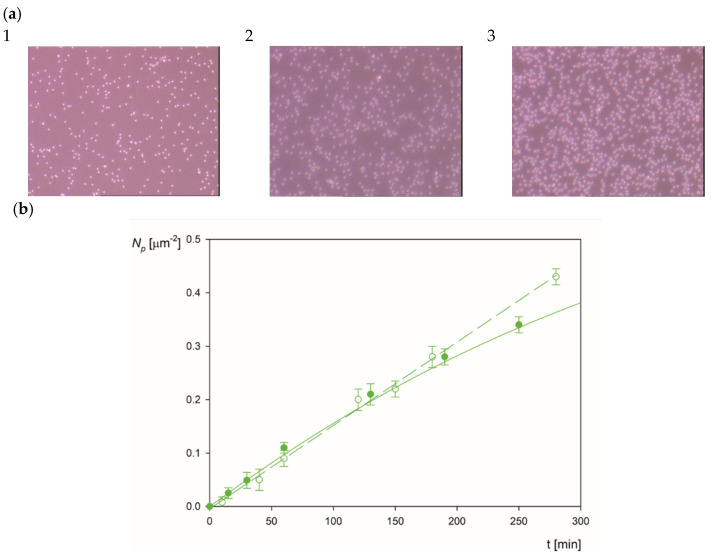
Part (**a**). Micrographs of the LSFi particles on mica for various surface concentrations equal to: 1—0.05 µm^−2^ (scale 135 µm × 100 µm), 2—0.2 µm^−2^ (scale 59 µm × 45 µm), 3—0.4 µm^−2^ (scale 59 µm × 45 µm) Part (**b**). The kinetics of LSFi particle deposition at mica in the OBIJ flow cell, shown as the dependence of the surface concentration on the deposition time, particle bulk concentration 100 mg L^−1^, pH 3.5, 0.001 mol L^−1^ NaCl, flow rate 2.5 × 10^−3^ cm^3^ s^−1^. The hollow points show the reference results acquired by in situ optical microscopy for positively charged LA microparticles, and the full points represent the results for LSFi particles. The solid line shows the theoretical results derived from the convective diffusion model with the RSA blocking function, and the dashed line represents the linear deposition regime.

After establishing a robust procedure for preparing polymer particles with well-defined corona coverage, an extensive series of experiments was performed with the aim to determine the pH range where the LSFi particles can be deposited in a non-specific way on bare and PLL modified mica. These experiments were carried out in the microfluidic OBIJ cell (see Supporting Information), which facilitates in situ and real-time observation of deposited particles using optical microscopy (the particle micrographs at various coverage are shown in Figure 5a). This enables the determination of the surface concentration of particles according to the above-described method, expressed for the sake of convenience as per one µm^2^.

In this way, the particle deposition kinetics, expressed as the dependence of *N_p_* on the time *t*, can be quantitatively determined for various pHs. Additionally, the stability of the particle layers under various physicochemical conditions can be acquired by performing controlled desorption experiments carried out in situ under various flow rates (Supporting Information).

The results of the deposition kinetic experiments performed according to the above procedure for the LSFi particles at pH 3.5 and 0.001 mol L^−1^ NaCl are shown in Figure 5b. It should be mentioned that under these conditions, the zeta potential of the particle is equal to 36 mV and the zeta potential of mica is equal to −45 mV. It can be seen that the LSFi particle surface concentration *N_p_* linearly increases with the time below 200 min (see the dashed line in Figure 5b), which can be described by the formula

(2) $$N_{p}=k_{c}n_{p}t$$

where *k_c_* is the mass transfer rate constant in the cell and $n_{p}=\frac{6}{\pi d^{3}\rho_{p}}c_{p}$ is the particle number concentration in the suspension *d_p_* is the particle diameter and *ρ_p_* is their density.

Interestingly, an analogous linear kinetics was experimentally determined for the positively charged amidine particles (marked by hollow points in Figure 5b). Thus, for both the LSFi and the amidine particles the linear deposition regime is characterized by the mass transfer rate constant equal to 8.0 × 10^−6^ cm s^−1^, denoted as *k*_*c*0_. This constant is used as a scaling variable for the analysis of LSfi particle deposition kinetics at other pHs.

The entire kinetic run shown in Figure 5b was theoretically interpreted in terms of the hybrid approach described in the Supporting Information. Accordingly, in this model, the bulk transport of particles is described by the convective diffusion with the blocking effects quantitatively accounted for by the random sequential adsorption (RSA) approach [24]. One can observe in Figure 5b that the theoretical results obtained in this way adequately reflect the experimental data for the entire range of the deposition time, which indicates that the particle deposition attained the maximum value pertinent to the barrier-less transport conditions [25].

**Figure 6:**
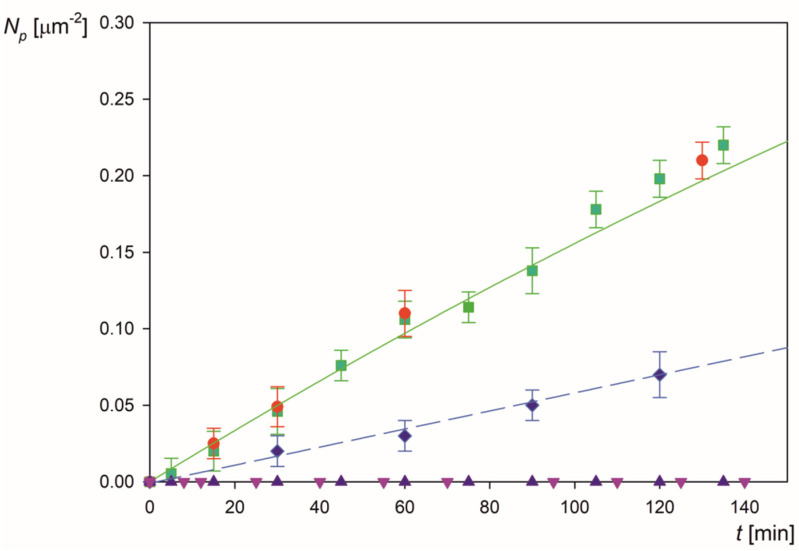
The kinetics of LSFi particle deposition at mica in the OBIJ flow cell, shown as the dependence of the surface concentration on the deposition time, particle concentration 100 mg L^−1^, 0.001 mol L^−1^ NaCl; flow rate 2.5 × 10^−3^ cm^3^ s^−1^. The points show the results obtained for various pHs: pH 3.5 (●); pH 4 (■), pH 4.5 (♦); pH 5.5 (▲); pH 7.4 (▼); The solid line shows the theoretical results derived from the convective diffusion model with the RSA blocking function.

Analogous LSFi particle deposition kinetic runs were performed for larger pHs, and the results are shown in Figure 6. It is seen that the deposition kinetics is practically constant at pH up to 4.5. However, for larger pHs, the adsorption kinetics abruptly decreases and is characterized by a practically negligible rate. These results were quantified by introducing the normalized particle deposition efficiency, which can be expressed under the linear regime by the formula.

(3) $$\bar{k}=k_{c}(pH)/k_{c0}$$

where *k_c_*(pH) is the mass transfer rate, calculated as the slope of the experimental dependence of *N_p_/n_p_* on the deposition time, experimentally determined for a given pH.

**Figure 7:**
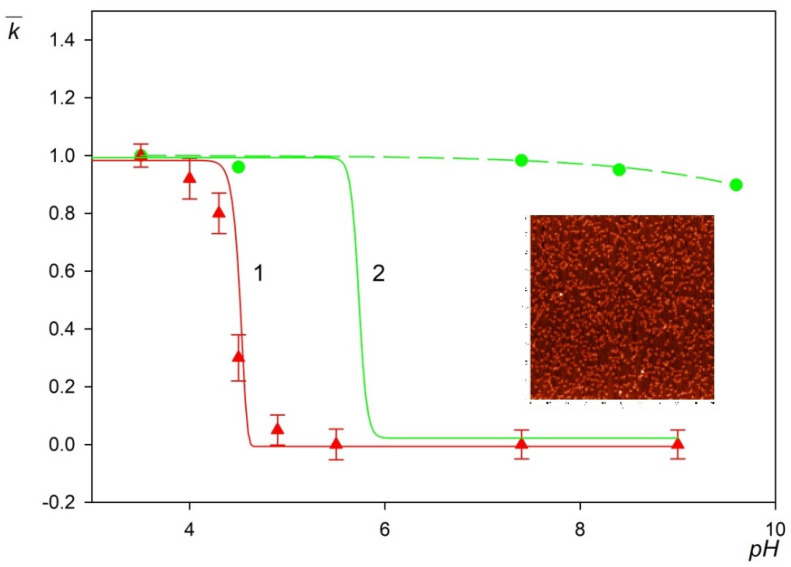
The normalized deposition rate $\bar{k}$ of LSfi particles (triangles, optical microscopy) and fibrinogen molecules (circles, AFM) at mica as a function of pH (NaCl concentration of 0.01 mol L^−1^). The solid lines 1 and 2 show the theoretical results calculated from the mean-field approach for the particles and fibrinogen, respectively, and the dashed line is a fit of the experimental data for fibrinogen. The inset shows the fibrinogen layer at mica imaged by AFM (scale 1 µm × 1 µm).

The experimental results obtained for the LSFi particles normalized in this way are shown in Figure 7 for pH range from 3.5 to 9 (triangles). As can be seen, for pH above 4.5, the deposition rate of the particles abruptly decreases to negligible values. This behavior well correlates with the decrease in the zeta potential of the particles, which becomes negative for pH larger than 4.5, i.e., of the same sign as the zeta potential of mica equal to −50 mV at this pH (see Figure 1b). In order to quantitatively analyze this effect, theoretical calculations were performed according to the procedure described in the Supporting Information. The exact mass transfer equation was numerically solved adopting the mean-field interaction potential consisting of the electrostatic component controlled by the zeta potential and the double-layer thickness as well as the van der Waals component. The solution of the transport equation directly yielded the mass transfer rate constant for a given pH that allowed to calculate the normalized deposition rate $\bar{k}$. One can observe in Figure 7 that the theoretical results derived in this way for the LSFi particles (shown as the solid line number 1), well reflect the experimentally observed trend, i.e., an abrupt decrease in the deposition rate with pH around 4.5. This agreement suggests that the deposition kinetics of the particles with fibrinogen corona at negatively charged substrates can be adequately analyzed in terms of the mean-field approach analogously to colloid particles [41].

However, a different situation occurs for the fibrinogen molecule adsorption kinetics at mica determined by AFM. The experimental protocol of AFM-based procedure was as follows. After deposition of the fibrinogen on mica sheets samples were dried and imaged using AFM under ambient conditions. The number of molecules adsorbed on mica was determined by direct counting procedure. As can be inferred from Figure 7, in his case, the normalized adsorption rate remains practically constant for pH up to 9, whereas the fibrinogen molecule zeta potential becomes negative at pH larger than 5.8. This is more evident comparing the experimental data for fibrinogen with the mean-field results shown by line number 2 in Figure 7. Such anomalous fibrinogen adsorption at pHs above its isoelectric point at negatively charged substrates was previously reported [22] and interpreted in terms of heterogeneous charge distributions.

One can argue that the experimental results shown in Figure 7 have important practical aspects because they confirm that at pH > 5, in particular at pH 7.4, a non-specific (physical interactions driven) deposition of the polymer particles with the fibrinogen corona becomes negligible. This creates the possibility for conducting efficient immunoassays for this pH range exploiting the specific antigen/antibody interactions.

### 2.4. Fibrinogen/Microparticle Complex Deposition at PLL Layers

**Figure 8:**
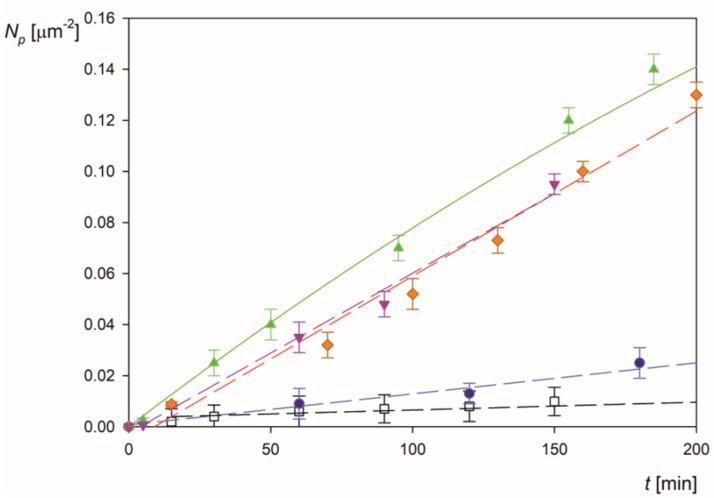
Kinetics of LSFi particle deposition at the mica/PLL substrate in the OBIJ flow cell, shown as the dependence of the surface concentration on the deposition time, particle concentration 50 mg L^−1^, 0.01 mol L^−1^ NaCl, flow rate 2.5 × 10^−3^ cm^3^ s^−1^. The points show the results obtained for various pHs: pH 9 (▼); pH 7.4 (♦); pH 6.5 (▲); pH 4.5 (●); pH 3.5 (□). The solid line shows the theoretical results derived from the convective diffusion model with the RSA blocking function.

Analogous LSFi particle deposition kinetics runs were performed for the PLL modified mica exhibiting positive zeta potential for pH up to 9 (see Figure 1a). Representative results obtained for various pHs and 0.01 mol L^−1^ NaCl concentrations are shown in Figure 8. One can observe that at pH 9 to 6, the particle deposition rate attains the maximum value pertinent to the barrier-less regime theoretically described by the RSA model (solid line in Figure 8). Afterward, for smaller pHs, the adsorption kinetics abruptly decrease and is characterized by a practically negligible rate. Analogously, as for bare mica, this behavior correlates with the zeta potential of the particles, which becomes positive at pH larger than 5, i.e., of the same sign as the zeta potential of PLL covered mica, equal to 40 mV.

It is interesting to mention that analogous deposition experiments of MS2 virus adsorption at PLL modified sensors were performed by Armanious et al. [39] using the quartz microbalance method. At pH 6 and 0.01 mol L^−1^ NaCl a substantial deposition of the virus was observed with the maximum coverage attaining 20 mg m^−2^.

**Figure 9:**
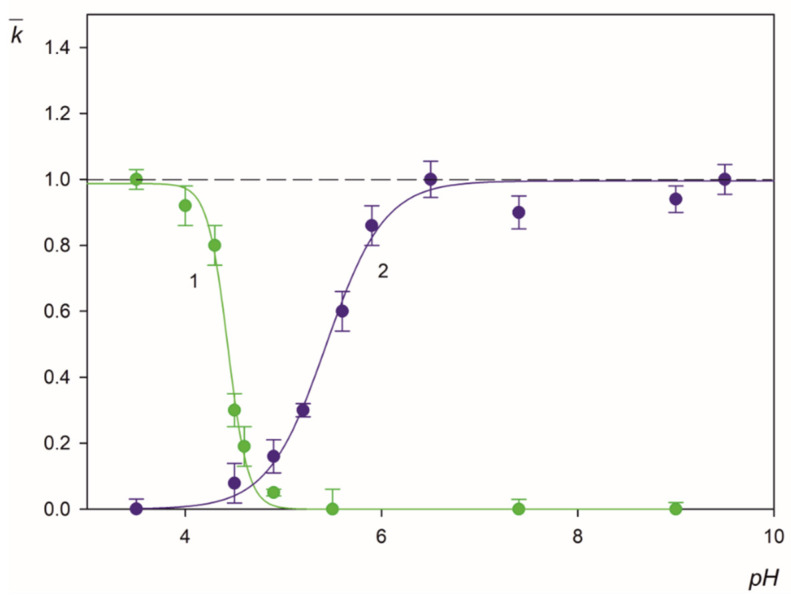
Normalized rate of LSfi particle deposition at mica and mica/PLL as a function of pH. The solid line 1 represents a fit of experimental data for mica/PLL deposition and solid line 2 is a fit of experimental data obtained for the mica/PLL substrate.

In Figure 9, the results obtained for the LSFi particles at PLL modified mica are shown as the dependence of the normalized deposition rate on pH and compared with previous results obtained for bare mica. One can summarize these results by specifying the pH window where the polymer particles with fibrinogen corona can only adsorb specifically. Hence, for negatively charged surfaces, this occurs at a pH larger than 5, and for positively charged surfaces, at pH smaller than 6. Therefore, by appropriately selecting the substrate either negatively or positively charged, the specific adsorption may occur at practically the entire pH range.

This information can be exploited for efficiently performing reliable, label-free immunological assays with a large sensitivity because of the direct detection of deposited particles via optical imaging.

## 3. Materials and Methods

### 3.1. Materials

Fibrinogen from human blood plasma, in the form of a crystalline powder containing 65% protein, 25% sodium chloride, and 15% sodium citrate, was supplied by Sigma and used without further purification. Fibrinogen solutions were prepared by dissolving an appropriate amount of the powder under gentle stirring at appropriate pH and 298 K in a sodium chloride solution. Afterward, the suspension was passed through the 0.45 μm filter and the bulk concentration of fibrinogen was spectrophotometrically determined using a Shimadzu UV-1800 Spectrophotometer. The concentrated solutions (typically 300–500 mg L^−1^) used for the electrophoretic mobility measurements) were diluted by a sodium chloride solution to the desired bulk concentration, usually 1–5 mg L^−1^ prior to each adsorption experiment. The pH in the range of 3–5 was adjusted by the addition of HCl, the pH of 7.4 was fixed by the PBS buffer, and larger pHs were adjusted by NaOH.

Water was purified using a Millipore Elix 5 apparatus. Chemical reagents (sodium chloride, hydrochloric acid) were commercial products of Sigma-Aldrich (Darmstadt, Germany) and were used without further purification.

The suspension of negatively charged sulfonate polystyrene microparticles used as colloid carriers for fibrinogen, referred to as LS, was our own product synthesized according to the Goodwin procedure [42]. The stock suspension of a concentration determined by densitometry and the dry mass method, was diluted to 50 to 100 mg L^−1^ in the fibrinogen corona formation and the particle deposition experiments. In addition, the suspension of positively charged amidine microparticles, referred to as LA, a product of ThermoFisher (Life Technologies, Warsow, Poland), was used for performing reference deposition measurements.

Ruby muscovite mica obtained from Continental Trade was used as a model substrate. The solid pieces of mica were freshly cleaved into thin sheets prior to each particle deposition experiment carried out in a diffusion cell under thermostated conditions.

Poly-L-lysine hydrobromide (PLL), a synthetic polypeptide having a molar mass of 75–189 kg mol^−1^ (determined by MALS), was purchased from Sigma Aldrich Merck KGaA, Darmstadt, Germany. The average molar mass of the PLL sample of 120 kg mol^−1^ was more precisely determined by dynamic viscosity measurements [43].

The modification of the mica substrate by PLL was carried out according to the previously described procedure [44]. Briefly, a few freshly cleaved mica sheets were vertically immersed in the PLL solution of a regulated concentration, pH, and ionic strength, typically 10 mg L^−1^, 5.6 and 0.15 mol L^−1^ NaCl. The adsorption was continued under pure diffusion conditions over 10 min in a thermostated cell. Afterward, modified mica was washed three times in water, and it was placed on the flow cell.

### 3.2. Experimental Methods

The zeta potential of bare and PLL modified mica sheets was determined by the streaming potential method using a microfluidic cell in the form of the parallel plate channel [44]. Initially, the streaming potential was measured using a pair of reversible electrodes as a function of the hydrostatic pressure difference Δ*P*. Subsequently, the streaming potential was converted to the zeta potential *ζ* using the Smoluchowski relationship [45].

The diffusion coefficient of fibrinogen, microparticles, and complexes was determined by the dynamic light scattering (DLS) using the Zetasizer Nano ZS instrument from Malvern (A.P. Instruments, Warsow, Poland). The hydrodynamic diameter was calculated using the Stokes–Einstein relationship. The electrophoretic mobility of fibrinogen and the particles was measured using the Laser Doppler Velocimetry (LDV)technique using the same apparatus. The zeta potential was calculated using the Henry model for fibrinogen or using the Smoluchowski model for particles.

The deposition kinetics of the particles with fibrinogen coronas at these two substrates was determined using the oblique impinging-jet cell (see Supporting Information) according to the previously described procedure [25]. A steady laminar flow of the suspension was generated by the hydrostatic pressure difference between two containers, which enabled the regulation of the volumetric flow rate within broad limits. It should be mentioned that because of the under-pressure prevailing in the cell, the mica substrate in the form of freshly cleaved sheets was firmly attached to the cell wall without using any adhesive. This eliminated the possibility of the contamination of the cell during the measurement. Deposited particles were observed in situ using an inverted optical microscope from Leica equipped with long-distance objectives, a camera, and imaging processing software. The number of particles per unit area (typically one square micrometer, denoted hereafter by *N_p_*) was determined by direct counting of over 10–20 equal-sized areas randomly chosen over the mica surfaces with the total number of particles exceeding 1000. This provides a relative precision for these measurements of more than 92%. Using the known values of the surface concentration *N_p_* the absolute (dimensionless) coverage of particles was calculated as *Θ* = *N_p_ S_g_*, where *S_g_* is the characteristic cross-section area of the particles.

The fibrinogen monolayers for AFM imaging were prepared ex-situ under diffusion-controlled transport conditions. Freshly cleaved mica sheets were immersed in 1 mg L^−1^ protein solution of constant ionic strength of 0.01 mol L^−1^ NaCl and desired pH (3.5–9.5) for 15 min, then were rinsed with ultrapure water and dried in a stream of nitrogen.

The dry samples of fibrinogen-covered mica were imaged under ambient conditions using the NT-MDT Solver PRO (Russia) device with the SMENA SFC050L scanning head. The measurements were performed in semi-contact mode using silicon probe (golden silicon cantilevers with resonance frequencies of 47 kHz +/− 10% to 150 kHz +/− 10%, a typical tip curvature of 10 nm, and a cone angle less than 20°).

The temperature of the experiments was kept at a constant value equal to 298 ± 0.1 K.

### 3.3. Theoretical Modeling

Adsorption of fibrinogen on polymer particles was theoretically modeled applying the random sequential adsorption (RSA) approach described in Supporting Information. This is a stochastic process in which objects (protein molecules) are consecutively placed at a surface in such a way that they do not overlap any previously adsorbed particles [46,47,48,49]. Another necessary condition is that they can only irreversibly adsorb after contacting an uncovered surface area of the interface. By virtue of these assumptions, the adsorption process is completed when there are no available (uncovered) collector areas. The coverage attained in this limit is referred to as the jamming coverage and represents the most relevant parameter determined in RSA modeling.

In previous works [9,10,22], the RSA approach was applied to model the adsorption of fibrinogen on flat substrates and at polymer microparticles of negative charge. In these calculations, the coarse-grained model was adopted where the real shape of the fibrinogen molecule was replaced by a string of co-linear touching spheres of various diameters with the length of the core part of the molecule equal to 48.7 nm (see Supporting Information). The presence of the Aα chains is approximated as a straight sequence of *n_s_* beads of equal size, and they are assumed to form the angle *φ* with the main core axis. Both the number of beads *n_s_* and the angle *φ* can be varied, which assures higher flexibility of theoretical calculations [33].

Initially, in the soft–RSA modeling, the charge distribution on the fibrinogen molecule was generated with the total number of charges experimentally determined via electrophoretic mobility measurements [9,33]. For this charge distribution, the electrostatic interactions of the adsorbing fibrinogen molecule with the molecules attached to the surface were calculated using the Yukawa pair energy, physically derived from the screened Coulomb interactions

(4) $$\phi_{12}=\;\frac{e^{2}}{4\pi \;\varepsilon r_{12}}\;e^{\;-\kappa (r_{12}-a_{1}-a_{2})}$$

where *e* is the elementary charge, *r*_12_ is the distances between the centers of the two beads of the radii of *a*_1_ and *a*_2_, belonging to the adsorbing and the adsorbed fibrinogen molecules, $\kappa^{-1}={\left(\frac{\varepsilon kT}{2e^{2}I}\right)}^{1/2}$ is the electrical double-layer thickness, *ε* is the permittivity of the medium, *k* is the Boltzmann constant, and *I* is the ionic strength of the electrolyte solution. Using the pair potential, Equation (4), one can express the interaction energy of the adsorbing molecule with the *l*-th adsorbed molecule $\phi_{al}$ in the following form [41]

(5) $$\phi_{al_{}}=\sum_{i=1}^{i_{mx}}\sum_{j=1}^{i_{mx}}\phi_{aijl}$$

where *ϕ_aijl_* is the pair energy of the *i*-th bead of the adsorbing molecule with the *j*-th bead of the *l*-th molecule in the interaction zone and *i_mx_* is the total number of beads. Consecutively, the net interaction energy of the adsorbing molecule with adsorbed molecules, denoted by $\phi_{a}$ was calculated by summing up their interactions with the adsorbed fibrinogen molecule located within the interactions zone.

(6) $$\phi_{a}=\sum_{l=1}^{N_{i}}\phi_{al}$$

where *N_i_* is the number of molecules in the interaction zone. Finally, the probability density of fibrinogen molecule adsorption at a given point at the interface was calculated from the Boltzmann formula:

(7) $$\rho_{p_{v}}=e^{\;-\phi_{a}/kT}$$

Using this algorithm, three various adsorption regimes were efficiently modeled (see Supporting Information): (i) the exclusively side-on regime, (ii) the end-on regime, and (iii) the mixed regime where the molecules can adsorb in the end-on orientation if there is not enough space for the side-on orientation. The most relevant quantities derived from the modeling were the numbers of adsorbed fibrinogen molecules forming the corona in the side-on *N_p_*_║_ and the end-on $N_{p⊥}$ orientations [9]. Knowing these numbers, the net surface concentration of molecules can be calculated as

(8) $$N=(N_{p}{}_{║}+N_{p⊥})/S_{p}$$

where $S_{p}=\pi d_{p}^{2}$ is the geometrical area of the polymer particle of the diameter *d_p_*. Consequently, the mass coverage of fibrinogen on the particle is given by

(9) $$\Gamma =\frac{M_{w}}{Av}N_{}$$

where *Av* is the Avogadro constant and *M_w_* is the molar mass of fibrinogen. Ten independent runs were performed, which gives the overall number of fibrinogen molecules equal to 5 × 10^4^. This yields the relative error of the maximum coverage determination smaller than 0.5%.

On the other hand, the deposition kinetic of the particles at the flat substrates under flow conditions was quantitatively modeled in terms of a hybrid approach. Accordingly, the bulk transport was described by the convective–diffusion equation coupled with the surface layer transport equation where the convection effect was neglected, serving as the boundary condition (Supporting Information).

This approach yields the following implicit expression for the particle number concentration as a function of deposition time *t*

(10) $$\int_{0}^{\Theta }\frac{\;\;\;\;\;(k_{a}-k_{c})\;B(\tilde{\Theta })+k_{c}}{k_{a}k_{c}\;S_{g}n_{b}B\left(\tilde{\Theta }\right)-k_{d}k_{c}\tilde{\Theta }}\;\;d\tilde{\Theta }=t$$

where $\Theta =N_{p}\pi d_{p}^{2}/4$ is the absolute (dimensionless) coverage of the particles, *k_a_*, *k_d_* are the kinetic adsorption and desorption constants, and *k_c_* is the mass transfer constant.

In the case of oblique impinging jet flow, the mass transfer rate constant is given by the analytical expression [24,25]

(11) $$k_{c}=0.776\frac{\alpha_{r}^{1/3}V_{\infty }^{\;1/3}\;D^{2/3}}{r_{b}{}^{2/3}}\;=0.530\frac{\alpha_{r}^{1/3}Q^{1/3}\;D^{2/3}}{r_{b}{}^{4/3}}$$

where *α_r_* is the dimensionless parameter depending on the flow Reynolds number *Re*, *V_∞_* is the characteristic liquid velocity in the jet, *Q* is the volumetric flow rate, *D* is the diffusion coefficient of the particles, and *r_b_* is the characteristic dimension of the jet.

## 4. Conclusions

An efficient method for the preparation of stable suspensions of polymer particles with a fibrinogen molecule corona was developed. Using the electrophoretic mobility and concentration depletion methods based on AFM, the coverage and molecule orientation in the coronas were determined. The experimental results agreed with the theoretical data derived from the coarse-grained random sequential adsorption modeling.

It is also confirmed in these experiments that the zeta potential of particles is strictly correlated with the bulk zeta potential of fibrinogen and the corona coverage. Therefore, it is argued that this regularity is also valid for coronavirus zeta potentials. This is especially important for the SARS-Cov-2 virus, where the spike protein zeta potential can be experimentally determined for its recombinant version, whereas for the intact virion the zeta potential measurements are impractical.

Adequate stability of the particles with fibrinogen coronas also allowed to carry out thorough deposition kinetics measurements in the microfluidic impinging-jet cell both for negatively and positively charged substrates. It is shown that in the case of the negatively charged substrate (mica), the deposition kinetics of particles vanished at pH above 5. This effect was quantitatively accounted for by the mean-field approach, in analogy to the colloid particle deposition. This is in contrast to the fibrinogen molecule behavior, which efficiently adsorb at negatively charged substrates for the entire range pHs up to 9.7.

These results have practical significance demonstrating that at pH above 5, in particular at pH 7.4, the physical interaction-driven deposition of the particles with fibrinogen corona on negatively charged surfaces becomes negligible. This creates the possibility for conducting efficient label-free immunoassays governed by the specific antigen/antibody interactions.

## Data Availability

The data is available on request.

## Supplementary Materials

The following are available online. Figure S1: The fibrinogen coronas at LS polymer particles (820 nm in diameter) derived from the RSA modeling for various adsorption regimes (0.01 mol L^−1^ NaCl, pH 3.5):1-side-on adsorption, *N* = 990 µm^−2^ (*Γ* = 0.56 mg m^−2^), 2-end-on adsorption, *N* = 5700 µm^−2^ (*Γ* = 3.2 mg m^−2^), 3-side-on/end-on adsorption simultaneously, *N* = 5100 µm^−2^ (*Γ* = 2.8 mg m^−2^), the side-on and end-on adsorbed molecules are marked in red and blue color, respectively. Figure S2: The stability of the LSFi particle suspension (100 mg L^−1^, 0.01 mol L^−1^ NaCL) at pH 3.5 (▲) and pH 7.4 (●) (PBS) expressed as the dependence of the normalized hydrodynamic diameter d_H_/d_H0_ and the normalized zeta potential ζ/ζ_0_ on the storage time (where and d_H0_ are the zeta potential and the hydrodynamic diameter for the initial time). Figure S3: The microfluidic OBIJ cell: 1. the container with the particle suspension, 2. the inlet tubing with the capillary, 3. the transparent substrate plates, 4. the inverted optical microscope, 5. the outlet tubing, 6. the used suspension container. Adapted from Reference [12]. Figure S4: The kinetics of LSFi particle deposition/desorption at mica in the OBIJ flow cell, shown as the dependence of the surface concentration on the deposition time, pH 3.5, 0.001 mol L^−1^ NaCl, particle concentration 100 mg L^−1^, flow rate 2.5 × 10^−3^ cm^3^ s^−1^. At the time of 300 min, the desorption run was initiated where the pure electrolyte of the same pH and flow rate was flushed through the cell. The solid line is the fit of experimental data. Figure S5: The desorption kinetics of the LSFi particles under determination for the OBIJ cell (0.01 mol L^−1^ NaCl, volumetric flow rate 2.5 × 10^−3^ cm^3^ s^−1^) expressed as the dependence of the normalized surface concentration N_p_/N_p0_ on the desorption time: pH 3.5 (▲), pH 7.4 (●). Table S1: Model shapes of the fibrinogen molecule, from Reference [5].

## Sample Availability

Tested samples are available from the corresponding author (P.Ż.) on request.
