# Supplementary material for: Deposition of Polymer Particles with Fibrinogen Corona at Abiotic Surfaces under Flow Conditions
Source: Molecules. 2021 Oct 18;26(20):6299. doi: 10.3390/molecules26206299 (PMC8538388; doi:10.3390/molecules26206299)
Supplement: Supplementary file 1 [file molecules-26-06299-s001.zip › molecules-1399565-supplementary.pdf]

Deposition of Polymer Particles with Fibrinogen Corona at Abiotic Surfaces under Flow Conditions

Paulina Żeliszewska[1]*, Monika Wasilewska[1], Michał Cieśla[2], Zbigniew Adamczyk[1] *

[1]*Jerzy Haber Institute of Catalysis and Surface Chemistry Polish Academy of Science, Niezapominajek 8, 30-239 Kraków, Poland.*

[2]*Jagiellonian University, Faculty of Physics, Astronomy, and Applied Computer Science, Stanisława Łojasiewicza 11, 30-348 Krakow, Poland*

*Corresponding author,

zbigniew.adamczyk@ikifp.edu.pl
paulina.zeliszewska@ikifp.edu.pl

1. Modeling of fibrinogen adsorption kinetics using the soft RSA approach

Adsorption of fibrinogen molecules on polymer particles was theoretically modeled applying the random sequential adsorption (RSA) approach. This is a stochastic process where particles (for example protein molecules) are placed consecutively at a surface in such a way that they do not overlap any previously adsorbed ones [1-4]. Another necessary condition is that the particles can only adsorb after contacting with an uncovered surface area of the interface. Upon adsorption, the particles remain immobilized under a give position and cannot desorb. By virtue of these assumptions the adsorption process is completed when there is no available (uncovered) interface area large enough to accommodate the adsorbing particle. The coverage attained in this limit is referred to as the jamming coverage and represents the most relevant parameter determined in RSA modeling.

In this work, fibrinogen adsorption was analyzed using a coarse-grained, bead Model B of the fibrinogen molecule (see Table 1) where the presence of the side chains is explicitly taken in to account [5,6]. In terms of this model, the real shape of the molecule is replaced by a string of 23 co-linear touching spheres of various diameters. The two external spheres have the diameters of 6.7 nm and the central sphere has a diameter of 5.3 nm. The remaining 20 spheres of an equal size have the diameter of 1.5 nm. Therefore, the length of the core part of the molecule is equal to 48.7 nm. The side arms are modeled as a straight sequence of n_s beads of equal size, having the diameter of d_4. In calculations performed in this work the number of the beads in the side chains was equal to 12 (see Table 1).

Table S1. Model Shapes of the Fibrinogen Molecule, from Ref. [5].

Model	Shape of Molecule	Remarks
Chemical	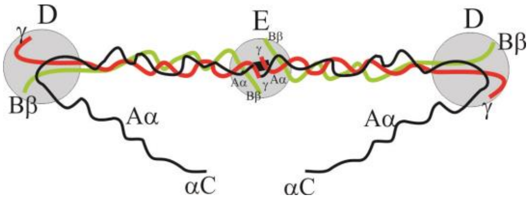	M_w = 337,897
Crystallographic	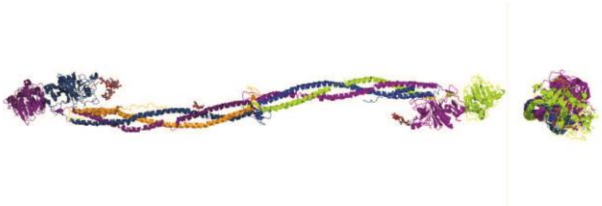	$\bar{v} = 0.72$ cm^3 g^{-1}<br>ρ = 1.38 g cm^{-3}<br>v = 405 nm^3
Bead Model B	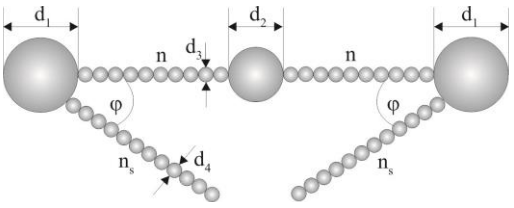	n = 10, n_s = 12<br>d_1= 6.7 nm, d_2 = 5.3 nm, d_3 = d_4 = 1.5 nm

A more general RSA model was adopted in the modeling, referred to as soft-RSA, where the electrostatic interactions of the adsorbing molecule with those attached to the interface were considered. Accordingly, in the first step in the soft-RSA modeling, a discrete charge distribution over the fibrinogen molecule was generated with the total number of charges experimentally determined via electrophoretic mobility measurements [6]. For this charge distribution, the electrostatic interactions of the adsorbing fibrinogen molecule with the molecules attached to the surface were calculated using the Yukawa pair potential, physically derived from the screened Coulomb interactions

$$\phi_{12} = \frac{e^2}{4\pi\,\varepsilon r_{12}} e^{-\kappa(r_{12}-a_1-a_2)} \qquad \text{(S1)}$$

where e is the elementary charge, r_{12} is the distances between the centers of the two beads of the radii of a_1 and a_2, belonging to the adsorbing and the adsorbed fibrinogen molecules, $\kappa^{-1} = \left(\frac{\varepsilon kT}{2e^2 I}\right)^{1/2}$ is the electrical double-layer thickness, ε is the permittivity of the medium, k is the Boltzmann constant, T is the absolute temperature, and I is the ionic strength of the electrolyte solution.

Using the pair potential, Eq. (S1), one can express the interaction energy of the adsorbing molecule with the l-th adsorbed molecule ϕ_{al} in the following form [7]:

$$\phi_{al} = \sum_{i=1}^{i_{mx}} \sum_{j=1}^{i_{mx}} \phi_{aijl} \tag{S2}$$

where ϕ_{aijl} is the pair energy of the i-th bead of the adsorbing molecule with the j-th bead of the l-th molecule in the interaction zone and i_{mx} is the total number of beads.

Consecutively, the net interaction energy of the adsorbing molecule with adsorbed molecules, denoted by ϕ_a was calculated by summing up their interactions with the adsorbed fibrinogen molecule located within the interactions zone

$$\phi_a = \sum_{l=1}^{N_i} \phi_{al} \tag{S3}$$

where N_i is the number of molecules in the interaction zone.

Finally, the probability density of fibrinogen molecule adsorption at a given point at the interface was calculated from the Boltzmann formula:

$$\rho_{p_v} = e^{-\phi_a / kT} \tag{S4}$$

Primarily, this soft–RSA modeling scheme yields the dependence of the number of adsorbed molecules N on the total number of attempts N_{att}. Then, using this dependence the maximum number of fibrinogen molecules adsorbed under the jamming state was precisely calculated using the interpolation procedure used in Ref.[8]. The method is based on the use of the asymptotic form of the equation describing the particle adsorption in the limit of the large number of attempts

$$N_{mx} = N_L + C_N N_{att}^{-1/q} \tag{S5}$$

where N_{mx} is the maximum number of adsorbed molecules for the infinite number of attempts, N_L is the surface concentration for a large (but finite) number of attempts and C_N, q are parameters determined from the RSA modeling.

Eq.(S5) can be used for efficient extrapolation of results obtained for the large number of attempts to the infinite number of attempts, impractical to attain.

Using this algorithm the fibrinogen adsorption at the polymer particles was modeled for the three adsorption regimes: (i) the side-on regime, (ii) the end-on regime and (iii) the mixed regime where the molecules can adsorb in the end-on orientation if there is not enough space for the side-on orientation.

The most relevant quantities derived from the modeling were the number of adsorbed fibrinogen molecules forming the corona in the side-on $N_{p\|}$ and the end-on $N_{p\perp}$ orientations after a given number of attempts. Knowing these numbers, the net surface concentration of molecules can be calculated as

$$N = (N_{p\|} + N_{p\perp}) / S_p \qquad \text{(S6)}$$

where $S_p = \pi d_p^2$ is the geometrical area of the polymer particle of the diameter d_p.

Snapshots of fibrinogen layers at the polymer particles of the size 820 nm, for 0.01 M NaCl concentration and pH 3.5, are shown in Fig. S1.

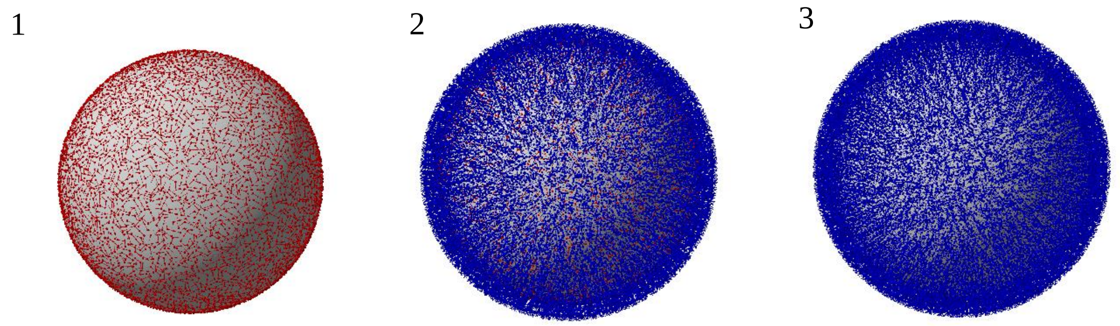

Fig. S1 The fibrinogen coronas at LS polymer particles (820 nm in diameter) derived from the RSA modeling for various adsorption regimes (0.01 M NaCl, pH 3.5):
1. side-on adsorption, N = 990 μm^{-2} (Γ=0.56 mg m^{-2})
2. end-on adsorption, N = 5700 μm^{-2} (Γ=3.2 mg m^{-2})
3. side-on/end-on adsorption simultaneously, N = 5100 μm^{-2} (Γ=2.8 mg m^{-2})

the side-on and end-on adsorbed molecules are marked in red and blue color, respectively.

The mass coverage of the fibrinogen corona at the particles can be calculated as

$$\Gamma = \frac{M_w}{Av} N \tag{S7}$$

where Av is the Avogadro constant and M_w is the molar mass of fibrinogen.

The absolute coverage of fibrinogen corona can be calculated as

$$\Theta = S_{gf} N \tag{S8}$$

where S_{gf} is the characteristic cross-section area of the fibrinogen molecule.

Ten independent runs were performed, which gives the overall number of fibrinogen molecules equal to 5×10^4. This yields the relative error of the maximum coverage determination smaller than 0.5%.

2. Calculation of the zeta potential of the particles with fibrinogen corona

The primary experimental data shown in Fig. 3 were interpreted in terms of the theoretical electrokinetic model formulated in Ref.[9,10]. In contrast to the Gouy-Chapmann approach, in this model three-dimensional fluid velocity and electric potential distributions around adsorbed protein molecules are considered in an exact way applying the multiple expansion method. This enabled the following expression for the zeta potential of interfaces covered by protein molecules $\zeta(\Theta)$ to be formulated

$$\zeta(\Theta) = F_i(\Theta)\zeta_i + F_p(\Theta)\zeta_p \tag{S9}$$

where ζ_i is the zeta potential of bare substrate, ζ_p is the particle (protein) zeta potential in the bulk, and $F_i(\Theta), F_p(\Theta)$ are the dimensionless functions. The F_i function describes the damping of the flow within the adsorbed molecule layer and the F_p function characterizes the contribution to the zeta potential stemming from the molecules. Accordingly, for low particle coverage, the F_i function approaches unity and the F_p function vanishes. For thin double-layers, one can express the functions by the following analytical expressions [10]

$$F_i(\Theta) = e^{-C_i \Theta}$$
$$F_p(\Theta) = a_p \Theta + b_p\left(1 - e^{-C_i \Theta}\right) \quad \text{(S10)}$$

where the C_i, a_p and b_p coefficients for spherical particles layers assume the limiting values of 10.2, 0.202 and 0.618, respectively.

For elongated particles in the form of touching bead strings, the C_i coefficients for various number of beads are given in Ref. [9].

Interestingly, Eq. (S10) is applicable for the random and equilibrium distributions of protein molecules in the coronas.

3. Convective- diffusion deposition of particles in the oblique impinging-jet (OBIJ) cell

Polymer particle deposition kinetics at solid substrates can be quantitatively described solving the continuity (mass balance) equation [11]:

$$\frac{\partial n}{\partial t} + \nabla \cdot \tilde{\mathbf{j}} = 0 \quad \text{(S11)}$$

where n is the number concentration of particles, t is the time and $\tilde{\mathbf{j}}$ is the generalized flux vector incorporating the translational and the rotary fluxes.

For spherical particles the rotational flux becomes irrelevant and the translation flux vector $\mathbf{j}$ can be expressed as:

$$\mathbf{j} = -\mathbf{D} \cdot \nabla n - \frac{1}{kT}\left(\mathbf{D} \cdot \nabla \phi\right) n + \mathbf{U}_h\, n \quad \text{(S12)}$$

where $\mathbf{D}$ is the translation diffusion tensor, $\mathbf{F} = -\nabla \phi$, is the interaction force of the particle with the substrate comprising the external and specific surface force contributions, ϕ is the net interaction potential, $\mathbf{U}_h = \mathbf{M} \cdot \mathbf{F}_h + \mathbf{M}_r \cdot \mathbf{To}_h$ is the particle velocity resulting from hydrodynamic forces $\mathbf{F}_h$ and torques $\mathbf{To}_h$.

Substituting the expression for the flux into Eq. (S11) one obtains the following continuity equation:

$$\frac{\partial n}{\partial t} = \nabla \cdot \left[\mathbf{D} \cdot \nabla n + \frac{1}{kT}\left(\mathbf{D} \cdot \nabla \phi\right) n - \mathbf{U}_h\, n\right] \quad \text{(S13)}$$

It should be mentioned that in Eq. (S13) the hydrodynamic and specific interactions among particles were neglected as well as the coupling between hydrodynamic and specific interactions. Moreover, the diffusion coefficient was assumed to be independent of the particle concentration.

It should be mentioned that exact solutions of Eq. (S13) for three-dimensional flows comprising the specific interactions term are not feasible by currently available computer software. However, useful solutions can be derived for some limiting forms of these equations having practical significance. The first one appears for uniformly accessible surfaces [11] where the perpendicular flow velocity component is independent of the tangential coordinate. This type of flow occurs for example for the rotating disk, and for the impinging-jet flows comprising the oblique impinging jet (OBIJ) flow applied in our experiments. In this case, Eq.(S13) becomes one-dimensional and assumes under the quasi-stationary conditions the following form [11]

$$\frac{d}{dH}\left[F_1(H)\left(\frac{d\bar{n}}{dH}+\frac{d\phi}{kTdH}\bar{n}\right)+\frac{1}{2}Pe\,F_1(H)F_2(H)(1+H)^2\,\bar{n}\right]=Pe\,F_3(H)(1+H)\bar{n} \quad \text{(S14)}$$

where H is the coordinate perpendicular to the interface scaled by the particle radius, $F_1(H),\ F_2(H),\ F_3(H)$ are the universal hydrodynamic correction functions, $\bar{n}$ is the scaled particle concentration, $Pe=\frac{V_{ch}\,d_p}{D_\infty}$ is the Peclet number characterizing the significance of the flow relative to the diffusion, V_{ch} is the characteristic velocity of the flow far from the interface and D_∞ is the diffusion coefficient of the particles in the bulk.

Numerical solution of Eq.(S14) acquired using the Runge-Kutta forth-order method yield the mass transfer rate constant k_c as a function of the flow rate and the particle diffusion coefficient. Also the influence of the energy barrier, described by the specific interaction potential ϕ consisting of electrostatic and van der Waals interactions, on the mass transfer rate can be adequately determined.

Accordingly, the surface concentration of deposited particles under this quasi-stationary transport regime described by Eq.(S14) is given by the linear dependence

$$N_p = k_c n_b t \tag{S15}$$

where n_b is the number concentration of the particles in the bulk and *t* is the deposition time.

However, the disadvantage of Eq.(S15) is that it is accurate for not too large surface concentration of particles where the surface blocking effects remain negligible.

The large coverage regime can be adequately treated in terms of the surface boundary layer approach [11] where the following kinetic equation describing particle transport near interfaces was formulated

$$\frac{dN_p}{dt} = k_c\,[n(\delta_a) - n_b] = k_a n(\delta_a) B\left(N_p\right) - k_d N_p \tag{S16}$$

where $n(\delta_a)$ is particle concentration at the surface boundary layer of the thickness δ_a, k_a, k_d are the kinetic adsorption and desorption constants, and $B\left(N_p\right)$ is the blocking function (referred more appropriately to as the available surface function [1-3]).

The adsorption and desorption constant can be calculated in an *ab initio* way if the particle/interface interaction potential is known using the expressions [9]:

$$k_a = \frac{e^{\phi(\delta_a)/kT}}{\displaystyle\int_{\delta_m}^{\delta_a} \frac{e^{\phi(z')/kT}}{D(z')} dz'} \tag{S17}$$

$$k_d = k_a e^{[\phi(\delta_m) - \phi(\delta_a)]/kT}$$

where $\phi(\delta_m)$ is the specific interaction energy of the particle at the primary minimum distance δ_m, $\phi(\delta_a)$ is the specific interaction energy of the particle with the interface evaluated at the distance δ_a and $D(z)$ is the diffusion coefficient of the particles, which depends on the distance from the substrate surface.

It should be mentioned that for a barrier-less adsorption regime and a deep primary minimum the desorption constant vanishes and the kinetic adsorption constant can be calculated from the dependence:

$$k_a = \frac{D}{\delta_a(1+0.5\ln\delta_a/\delta_m)} \tag{S18}$$

Under the convective diffusion transport where the mass transfer rate constant is independent of time one can express Eq.(S16) can be expressed in the form of the definite integral

$$\int_{\Theta_0}^{\Theta} \frac{(k_a - k_c)B(\tilde{\Theta}) + k_c}{k_a k_c\, S_g n_b B\left(\tilde{\Theta}\right) - k_d k_c \tilde{\Theta}}\, d\tilde{\Theta} = t \tag{S19}$$

where $\Theta = \pi d_p^2 N_p / 4$ is the absolute coverage of particles.

Eq.(S19) can be explicitly evaluated by conventional numerical methods which yields the particle deposition kinetics provided that the blocking function is known in an analytical form. This function can be conveniently acquired from the random sequential adsorption (RSA) modeling [1-3,11]. In the case of spherical particles, the exact numerical calculations were interpolated by the following analytical expression valid for the entire range of particle coverage [3]

$$B(\overline{\Theta}) = \left[1 + 0.812\overline{\Theta} + 0.4258\left(\overline{\Theta}\right)^2 + 0.0716\left(\overline{\Theta}\right)^3\right]\left(1-\overline{\Theta}\right)^3 \tag{S20}$$

where $\overline{\Theta} = \dfrac{\Theta}{\Theta_\infty}$ and Θ_∞ is the jamming coverage, equal to 0.547 for non-interacting (hard) particles of a spherical shape [1].

It was shown in Ref.[11] that the above results obtained pertinent to hard particles can also be extended to the case of particles interacting via the short- range Yukawa potential. For electrostatic double-layer interactions the characteristic range of this potential is given by [11]

$$h^* = \frac{1}{\kappa d_p}\left[\ln\frac{\phi_o}{\phi_{ch}} - \ln\left(1 + \frac{1}{\kappa d_p}\ln\frac{\phi_o}{\phi_{ch}}\right)\right] \tag{S21}$$

where ϕ_o is electrostatic energy at contact and ϕ_{ch} is the characteristic interaction energy.

Consequently, one can calculate the jamming coverage for interacting particles referred to as the maximum coverage) from the relationship

$$\Theta_{mx} = \Theta_{\infty} \frac{1}{(1+h^{*})^{2}} \qquad \text{(S22)}$$

Upon calculating Θ_{mx} one can use Eq.(S20) to calculate the blocking function substituting $\bar{\Theta} = \frac{\Theta}{\Theta_{mx}}$.

4. **Measurements of particle deposition kinetics in the OBIJ cell**

In preliminary experiments, the stability of the polymer particles with fibrinogen coronas was determined by measuring their diffusion coefficient and the electrophoretic mobility as a function of storage time. These data expressed as the dependence of the normalized zeta potential and the normalized hydrodynamic diameter on the storage time are shown in Fig. S2. As can be seen, the particles bearing the corona characterized by the coverage of 2.2 mg m^{-2} were stable over the time up to 24 hours, which suggests that they are prone to long-lasting electrokinetic investigations.

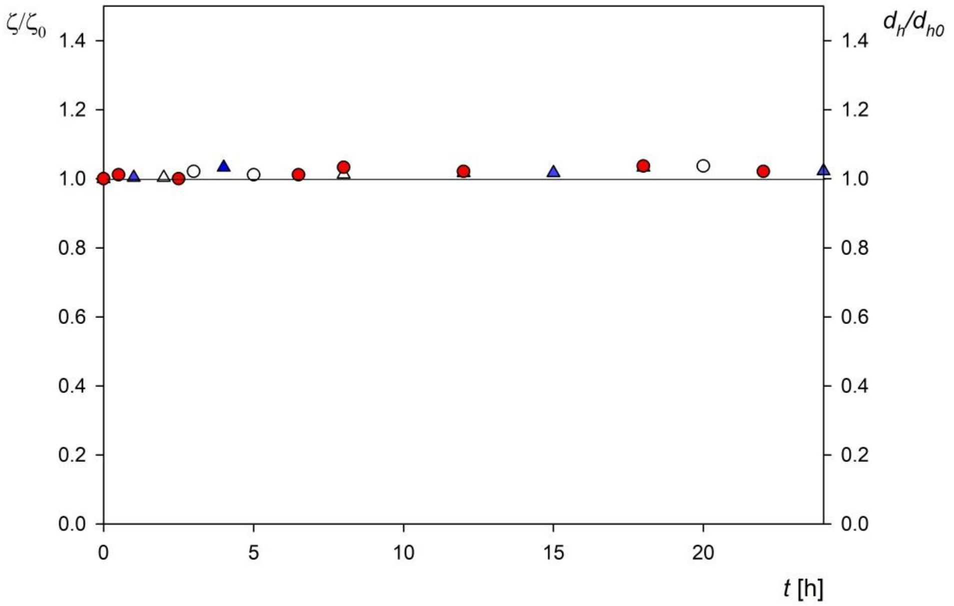

Fig. S2. The stability of the LSFi particle suspension (100 mg L^{-1}, 0.01 M NaCL) at pH 3.5 (▲) and pH 7.4 (●) (PBS) expressed as the dependence of the normalized hydrodynamic diameter d_H/d_{H0} and the normalized zeta potential ζ/ζ_0 on the storage time (where ζ_0 and d_{H0} are the zeta potential and the hydrodynamic diameter for the initial time).

The deposition kinetics of particles at bare and PLL modified substrates was determined using the oblique impinging-jet cell (see Fig. S3) according to the previously described procedure [12].

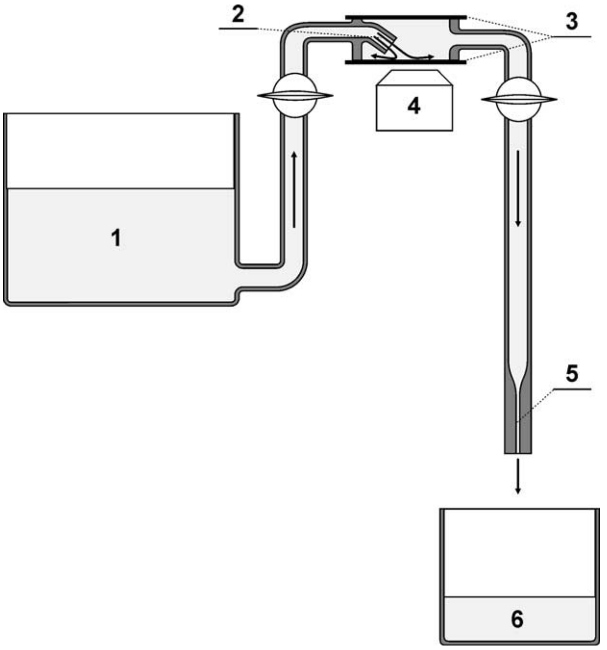

Fig. S3. The microfluidic OBIJ cell: 1. the container with the particle suspension, 2. the inlet tubing with the capillary, 3. the transparent substrate plates, 4. the inverted optical microscope, 5. the outlet tubing, 6. the used suspension container. Adopted from Ref. [12].

A steady laminar flow of the suspension was generated by the hydrostatic pressure difference between two containers 1 and 6, which enables to regulate the volumetric flow rate within broad limits. It should be mentioned that because of the under-pressure prevailing in the cell, the mica substrate in the form of freshly cleaved sheets was firmly attached to the cell wall without using any adhesive. This eliminated the possibility of the contamination of the cell during the measurement. Deposited particles were observed in situ using inverted optical microscope 4 equipped in long-distance objectives, camera, and imaging processing software. The number of particles per a unit area (typically one square micrometer, denoted hereafter by N) was determined by a direct counting of over 10-20 equal sized areas randomly chosen over the mica surfaces with the total number of number of particles exceeding 1000. This provides a relative precision of these measurements at more than 98%. Using the known values of the surface concentration N_p, the absolute

(dimensionless) coverage of particles was calculated as $\Theta = N_p\ S_g$, where S_g is the characteristic cross-section are of the particles.

The experimental run was completed by the desorption step. Accordingly, after completing the particle deposition run, where the pure electrolyte of a controlled pH and ionic strength was flushed through the cell at a regulated flow rate. The surface concentration of the particle monolayer was monitored in situ over a prolonged time in order to quantify the desorption rate. A primary particle deposition/desorption run acquired in this way is shown in Fig. S4. One can observe that the desorption of particles was negligible over the time of 300 min.

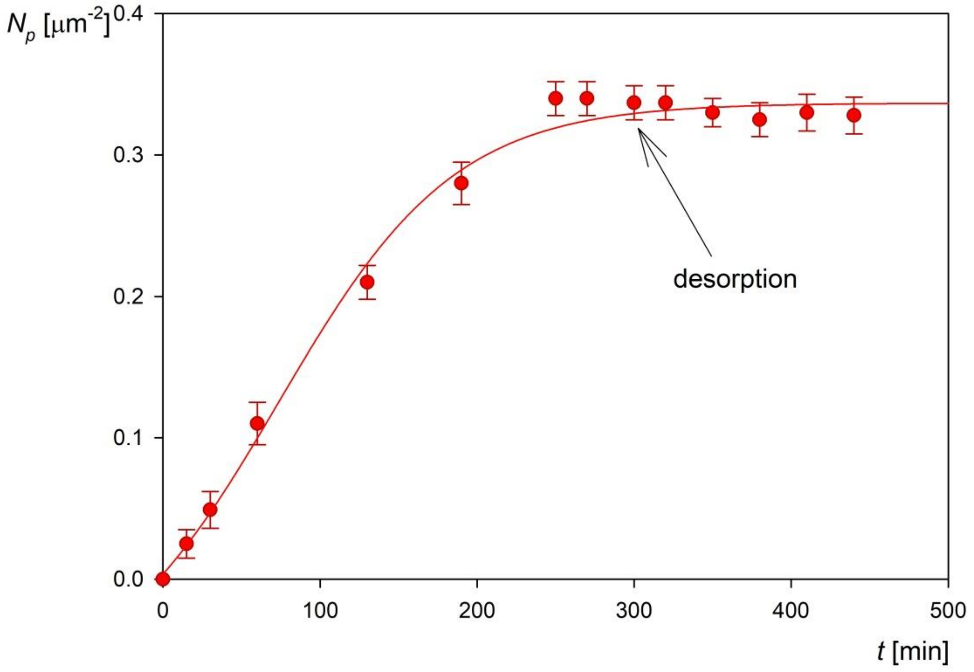

Fig. S4. The kinetics of LSFi particle deposition/desorption at mica in the OBIJ flow cell, shown as the dependence of the surface concentration on the deposition time, pH 3.5, 0.001 M NaCl, particle concentration 100 mg L^{-1}, flow rate 2.5×10^{-3} $cm^3\ s^{-1}$. At the time of 300 min. the desorption run was initiated where the pure electrolyte of the same pH and flow rate was flushed through the cell. The solid line is the fit of experimental data.

Analogous runs were performed for various surface concentrations of deposited particles where the ionic strengths and pH under the desorption run were different from those prevailing under the deposition run. For sake of convenience these results were expressed as the dependence of the normalized surface concentration of particles $N_p/\ N_{p0}$ (where N_{p0} is the initial surface concentration of particles after completing the deposition

run) on the desorption time. As shown in Fig. S5 the particle desorption at pH 3.5 and 7.4 was negligible.

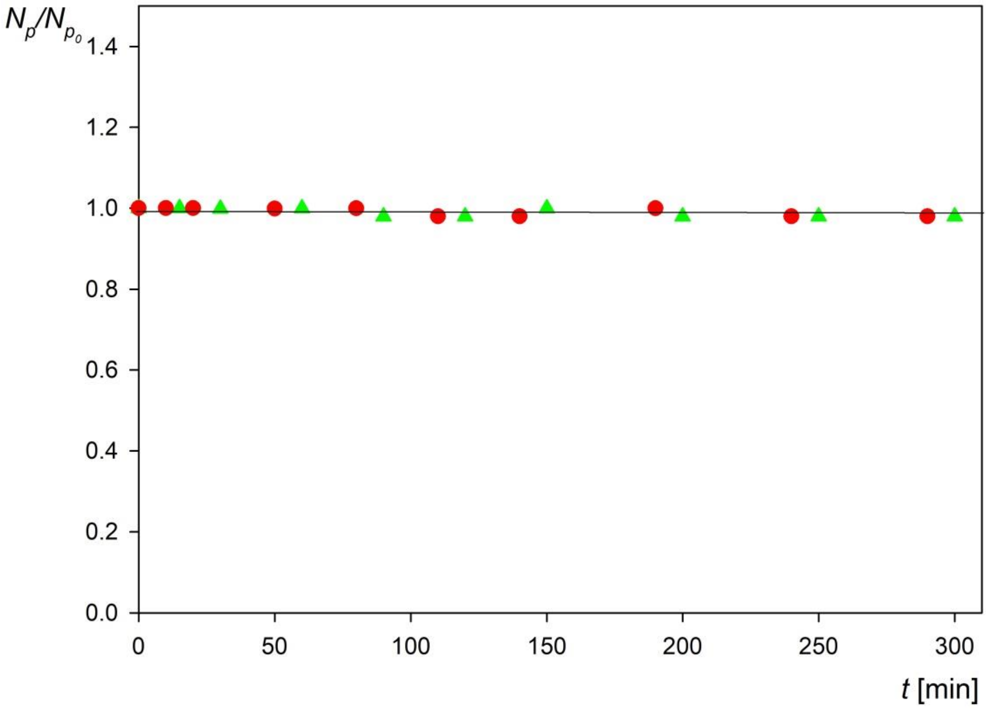

Fig. S5. The desorption kinetics of the LSFi particles under determined for the OBIJ cell (0.01 M NaCl, volumetric flow rate 2.5×10^{-3} cm^3 s^{-1}) expressed as the dependence of the normalized surface concentration N_p/ N_{p0} on the desorption time: pH 3.5 (▲), pH 7.4 (●) .

ACKNOWLEDGEMENTS

This work was financially supported by the Statutory activity of the J. Haber Institute of Catalysis and Surface Chemistry PAS. The authors are indebted to Katarzyna Kusak for her help in preparing the artwork.

References

[1] E. L. Hinrichsen, J. Feder, T. Jøssang, Geometry of random sequential adsorption, J. Stat. Phys. 44 (1986) 793-827.
[2] S.M. Ricci, J. Talbot, G. Tarjus, P. Viot, Random sequential adsorption of anisotropic particles. II. Low coverage kinetics, J. Chem. Phys. 97 (1992) 5219.
[3] J. Talbot, G. Tarjus, P.R. Van Tassel, P. Viot, From car parking to protein adsorption: an overview of sequential adsorption processes, Colloids Surf. Physicochem. Eng. Asp. 165 (2000) 287-324.
[4] M. Cieśla, Properties of random sequential adsorption of generalized dimers, Phys. Rev. E. 87 (2013) 52401-14.
[5] Z. Adamczyk, B. Cichocki, M. L. Ekiel – Jeżewska, A. Słowicka, E. Wajnryb, M. Wasilewska, Fibrinogen Conformations and Charge in Electrolyte Solutions Derived from DLS and Dynamic Viscosity Measurements, J. Colloid Interface Sci., 385 (2012) 244-57.
[6] A. Bratek-Skicki, P. Żeliszewska, Z. Adamczyk, M. Cieśla, Human Fibrinogen Monolayers on Latex Particles: Role of Ionic Strength, Langmuir 29 (2013) 3700-10.
[7] D. Kosior, M. Morga, P. Maroni, M. Cieśla, Z. Adamczyk, Formation of Poly-l-lysine Monolayers on Silica: Modeling and Experimental Studies, J. Phys. Chem. C 124 (2019) 4571-81.
[8] M. Cieśla, Z. Adamczyk, J. Barbasz, M. Wasilewska, Mechanisms of fibrinogen adsorption at solid substrates at lower pH, Langmuir 29 (2013) 7005-16.
[9] Z. Adamczyk, K. Sadlej, E. Wajnryb, M. Nattich, M.L. Ekiel-Jeżewska, J. Bławzdziewicz, Streaming potential studies of colloid, polyelectrolyte and protein deposition, Adv. Colloid Interface Sci. 153 (2010) 1-29.
[10] M.L. Ekiel-Jeżewska, Z. Adamczyk, J. Bławzdziewicz, Streaming current and effective ζ-potential for particle-covered surfaces with random particle distributions, J. Phys Chem. C 123 (2019) 3517-31.
[11] Z. Adamczyk, Particles at Interfaces: Interactions, Deposition, Structure, Elsevier, 2017.
[12] Z. Adamczyk, E. Musiał, B. Siwek, Kinetics of Particle Deposition in the Oblique Impinging Jet Cell, Colloid Interface Sci. 269 (2004) 53-61.
